# Sensing and Communicating β‐Cell Stress in the Context of T1D Etiology: New Opportunities for Therapeutic Impact

**DOI:** 10.1002/cph4.70198

**Published:** 2026-06-17

**Authors:** Diana Esparza, Chathurani S. Jayasena, Tijana Jovanovic‐Talisman, Debbie C. Thurmond

**Affiliations:** ^1^ Department of Molecular and Cellular Endocrinology Arthur Riggs Diabetes & Metabolism Research Institute, Beckman Research Institute at City of Hope Duarte California USA; ^2^ Department of Cancer Biology and Molecular Medicine Beckman Research Institute at City of Hope Duarte California USA

**Keywords:** β‐cells, double C2‐like domain containing beta protein (DOC2B), extracellular matrix (ECM), extracellular vesicles (EVs), immune cells, secretory pathway, type 1 diabetes (T1D)

## Abstract

Type 1 diabetes (T1D) has traditionally been viewed as an immune‐driven disease. However, evidence from pre‐onset T1D individuals suggests that pancreatic β‐cells show reduced metabolic gene expression and stress responses before substantial immune entry. In this review, we examine how chronically stressed β‐cells are detectable in a reshaped local microenvironment prior to overt immune cell infiltration, a period defined as a “pre‐immune” niche. During this period, pre‐onset β‐cells exhibit early extracellular matrix (ECM) remodeling capabilities, endoplasmic reticulum and Golgi stress, a shift in their soluble‐factor secretome and extracellular outputs, including release of extracellular vesicles with distinct cargo. Loss of the double C2‐like domain containing protein B (DOC2B), a regulator of vesicle trafficking and membrane fusion, may contribute to these processes. Beyond its canonical role in regulated insulin exocytosis, DOC2B negatively regulates cytokine‐induced CXCL10 expression in β‐cells via inhibition of IKKβ‐STAT1 signaling, and its loss increases activation of these pathways. DOC2B loss in cancer models promotes the formation of filopodia, protrusive structures capable of ECM engagement for matrix metalloproteinase‐mediated degradation; similarly, we consider whether changes in β‐cells could influence maladaptive interactions with the peri‐islet matrix during early T1D. Together, these concepts position DOC2B as a potential additional point of β‐cell vulnerability; further study may help guide early biomarker development and inform long‐term strategies for intercepting T1D before clinical onset.

## Graphical abstract

Type 1 diabetes (T1D) results from the immune‐mediated destruction of insulin‐producing pancreatic β‐cells. Recent human studies show that early β‐cell stress maladaptations precede overt immune cell infiltration. DOC2B, a vesicle secretion regulatory protein that supports β‐cell functionality and resilience against stress, declines early in T1D, promoting β‐cell dysfunction and immune cell access.

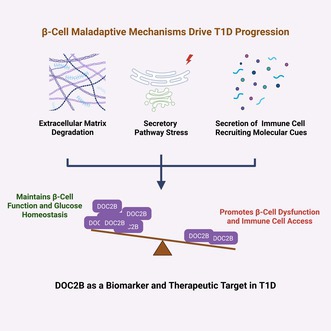

## 1 Introduction

Type 1 diabetes (T1D) is a debilitating disease characterized by the immune‐mediated destruction of insulin‐producing pancreatic β‐cells, leading to chronic hyperglycemia and lifelong insulin dependence. In 2025, an estimated 9.5 million people worldwide are living with T1D, a 13% increase since 2021. Alarmingly, 17.2% of T1D‐related deaths occur in individuals who remain undiagnosed even after symptoms appear (Ogle et al. 2025). These gaps in detection highlight a critical need to define mechanisms driving T1D progression from silent autoimmunity to clinical onset, with the aim of identifying sensitive biomarkers that inform on early β‐cell dysfunction.

Over the past decade, it has been increasingly clear that T1D heterogeneity extends beyond its autoimmune basis. While genetic susceptibility, particularly in the major histocompatibility complex (MHC) II region, confers significant risk, it accounts for only about half of the genetic contribution (Bauer et al. 2019). The presence of autoantibodies reactive to β‐cell antigens [e.g., insulin (IAA), glutamic acid decarboxylase (GAD65), tyrosine phosphatase‐like protein (IA‐2), and zinc transporter 8 (ZnT8)] currently serves as a predictor of disease progression, especially when multiple autoantibodies are present (Bauer et al. 2019). However, cohort studies reveal that progression varies widely depending on age, autoantibody type, and metabolic status (Ziegler et al. 2013; Bingley et al. 2018; Vehik et al. 2020). Insulitis, defined as the immune infiltration of pancreatic islets, is typically modest and heterogenous in humans, affecting only some insulin‐positive islets and varying by age and disease stage (Foulis et al. 1986; In't Veld et al. 2007; Willcox et al. 2009; Wiberg et al. 2015). Notably, insulitis alone does not cause diabetes (Higuchi et al. 1992; Picarella et al. 1993), raising the question whether β‐cells may initiate early disease processes rather than being passive targets of immune attack.

While β‐cell involvement in T1D pathogenesis is generally accepted (Atkinson et al. 2011; Roep et al. 2021), recent longitudinal data and cross‐sectional human studies indicate that β‐cell dysfunction can emerge early in disease, before destructive insulitis (Huber et al. 2025; Ferrannini et al. 2023). In single autoantibody‐positive donors, metabolic gene expression reductions can already be detected, and in T1D live pancreatic slices, β‐cell functional defects occur even in regions lacking local T‐cell infiltration (Huber et al. 2025). Consistent with these observations, early β‐cell stress or dysfunction has been reported in NOD mice before heavy insulitis (Engin et al. 2013; Lee et al. 2023; Postić et al. 2023; Mathisen et al. 2024). Together, these findings indicate that β‐cell abnormalities can precede widespread immune infiltration, underscoring the need to investigate early β‐cell intracellular and extracellular changes, and their downstream effects on the islet microenvironment in disease initiation.

A key downstream effect of early β‐cell stress is the remodeling of the local islet microenvironment. While short‐term stress responses are adaptive, prolonged activation becomes maladaptive; for example, β‐cells can promote degradation of extracellular matrix (ECM) components under autoimmune conditions (Johansen et al. 2025). In addition, chronic stress alters β‐cell inflammatory and secretory signaling, providing another route through which β‐cells could influence the islet niche and potentially increase immune accessibility.

Chronic stress can also impair components of the exocytosis machinery that governs glucose‐stimulated insulin secretion (GSIS) (Cheng et al. 2012; Aslamy, Oh, Ahn, et al. 2018; Amos et al. 2024; Bhowmick et al. 2025; Xie et al. 2025). This machinery includes the soluble N‐ethylmaleimide‐sensitive factor attachment protein receptor (SNARE) complex, composed of Syntaxin isoforms 1 and 4, SNAP25, and VAMP2, and regulatory proteins such as DOC2B [extensively reviewed in (Thurmond and Gaisano 2020)]. DOC2B levels decline under inflammatory and autoimmune stress, and this loss is associated with impaired β‐cell function, increased cytokine‐induced CXCL10 expression, and vulnerability to diabetogenic insults in mouse and cellular models (Aslamy, Oh, Ahn, et al. 2018; Bhowmick et al. 2025; Ramalingam et al. 2012; Aslamy, Oh, Olson, et al. 2018), although its specific role in autoimmune T1D remains to be fully elucidated. Nonetheless, these findings position DOC2B as a potential stress‐response marker and proximal regulator of β‐cell vulnerability.

In this review, we examine how chronically stressed β‐cells reshape their local microenvironment prior to overt immune cell infiltration, a stage defined as a “pre‐immune” niche (Huber et al. 2025). During this stage, pre‐onset T1D β‐cells exhibit early ECM remodeling capabilities (Johansen et al. 2025), endoplasmic reticulum (ER) and Golgi stress (Iida et al. 2023; Maestas et al. 2024; Mannering et al. 2005; Jin et al. 2011; McGinty et al. 2014; van Lummel et al. 2014; Xiang et al. 2015; Phelps et al. 2016; Bone et al. 2020; Isaacs et al. 2021), a shift in their soluble‐factor secretome and extracellular outputs, including release of extracellular vesicles (EVs) with distinct cargo (Pinheiro‐Machado et al. 2025; Sheng et al. 2011; Palmisano et al. 2012; Guay et al. 2015; Cianciaruso et al. 2017; Lakhter et al. 2018; Javeed et al. 2021; Dekkers, Lambooij, et al. 2024; Rao et al. 2025; Syed et al. 2026). Insights from cancer biology are informative here: in cancer models, reduced DOC2B expression is associated with epithelial‐to‐mesenchymal transition (EMT)‐linked cytoskeletal changes, including increased filopodia that support ECM engagement and invasive behavior (Bhat et al. 2022). Conversely, DOC2B enrichment reverses these phenotypes, and EVs released from DOC2B‐overexpressing cells carry cargo that suppresses filopodia formation in recipient cells (Bhat et al. 2022; Eswaran et al. 2025). Although DOC2B has not been studied in the context of ECM remodeling in β‐cells, studies in cancer cells raise the possibility that DOC2B loss could affect microenvironment‐related processes in stressed β‐cells. This hypothesis complements existing evidence that DOC2B deficiency increases β‐cell dysfunction and immunogenicity, highlighting DOC2B's relevance to early β‐cell stress in the context of T1D etiology.

## 2 Islet β‐Cells Contribute to Extracellular Matrix Degradation in T1D

### 2.1 The Islet Niche

The pancreas is 98%–99% comprised of exocrine cells, such as acinar cells, while the remaining 1%–2% are endocrine cells organized into species‐specific, three‐dimensional clusters, known as the islets of Langerhans (Kim et al. 2009). The bulk of islets consists of insulin‐producing β‐cells surrounded by glucagon‐producing α‐cells and somatostatin‐producing δ‐cells. Other lower abundant islet cell‐types include ghrelin‐producing ε‐cells, pancreatic polypeptide‐producing γ‐cells, endothelial cells lining intra‐islet capillaries, and resident immune cells. Islet architecture differs by species: in mice, β‐cells form a central core with α‐, δ‐, ε‐, and γ‐cells located at the islet periphery, whereas in humans and other primates, β‐cells are interspersed with other islet endocrine cells (Kim et al. 2009; Brissova et al. 2006; Cabrera et al. 2006).

A connective network of ECM proteins exists in islets: the interstitial matrix (IM) and the basement membrane (BM) (Irving‐Rodgers et al. 2008; Ziolkowski et al. 2012; Korpos et al. 2013). The IM occupies the space intermediately beneath the peri‐islet BM and extends through the intercellular region of islets. It is comprised of fibrillar collagens and fibronectin, among other components. BMs are primarily composed of collagen IV, laminins, heparan sulfate proteoglycans (HSPG), and hyaluronan (HA), enabling compartmentalization of islets from exocrine tissue and vasculature (Ziolkowski et al. 2012; Bogdani et al. 2014). In humans, the BM is double‐layered due to invagination of the peri‐islet membrane; consequently, β‐cells and α‐/δ‐cells are not in direct contact with vascular BM components (Virtanen et al. 2008). Together, the BM and IM provide islets immune privilege, conferring protection that affords islet cell viability and function. Disintegration of this protection underlies susceptibility and T1D progression, as is discussed in the following section.

### 2.2 The Islet Niche Disintegration in T1D

While the precise trigger for T1D in humans remains elusive, disease progression follows four distinct stages characterized by escalating immune engagement, as described in (Insel et al. 2015; Dayan et al. 2019; Atkinson and Mirmira 2023; Haller et al. 2024). Stage 1 involves the development of β‐cell autoimmunity, evidenced by the presence of at least two islet autoantibodies (IAA, GADA, IA‐2A, and ZnT8A), while normoglycemia is maintained. Stage 2 is characterized by persistent autoantibody positivity and emerging dysglycemia, indicated by impaired fasting glucose levels, abnormal glucose tolerance test, or glycosylated hemoglobin (HbA1c) ≥ 5.7%. Stage 3 culminates in symptomatic T1D onset, often presenting with polyurea, polydipsia, unexpected weight loss, fatigue, and sometimes diabetic ketoacidosis, when functional β‐cell mass has significantly declined. Stage 4 represents established T1D, characterized by insulin dependence and an increased risk of chronic complications, such as microvascular and macrovascular disease. Therefore, in this review, Stage 1 and Stage 2 will be regarded as the pre‐onset period.

Importantly, across these stages, immune cell passage through the islet ECM represents a critical mechanistic event, and loss of the peri‐islet ECM plays a pivotal role in T1D disease onset and progression (Irving‐Rodgers et al. 2008; Ziolkowski et al. 2012; Korpos et al. 2013; Simeonovic et al. 2018). Remodeling of the ECM (e.g., loss of collagen IV, laminin, nidogen, and perlecan) was reported in NOD mice at pre‐onset and in the pancreata from human donors with Stage 3 T1D (1–6 year disease duration) (Irving‐Rodgers et al. 2008; Korpos et al. 2013). Intriguingly, collagen IV loss has also been documented in autoantibody‐positive, pre‐onset human donors, indicating that selective ECM remodeling may begin before clinical onset in humans (Johansen et al. 2025). Metalloproteinases (e.g., gelatinases which breakdown collagen) are suspected to be involved in β‐cell ECM degradation (Yadav et al. 2003). Another point of vulnerability is the loss of HSPG in insulin‐positive islet cells, as observed in pancreatic tissue from individuals with Stage 3 T1D (Simeonovic et al. 2018). Indeed, treatment of NOD mice with heparinase inhibitor at pre‐onset led to preservation of heparan sulfate within islets and protected β‐cells from destructive autoimmunity and T1D (Ziolkowski et al. 2012). In addition, the accumulation of hyaluronan and the hyaluronan‐binding protein (HBP), including inter‐α‐inhibitor (IαI) and versican, not only occurs in T1D islets creating a permissive pathway for immune infiltration, but also correlates with the degree of insulitis (Bogdani et al. 2020). Altogether, these findings demonstrate that ECM composition serves a critical role in T1D immunity and accessibility of islet β‐cells.

In contrast to the autoimmune ECM remodeling described above, the islet niche undergoes fundamentally different structural and inflammatory changes in Type 2 diabetes (T2D). In T2D, peri‐islet fibrosis, driven by increased type I/III collagen, fibronectin, and hyaluronan, leads to global pancreatic stiffening, a mechanical environment that contributes to the paradoxical Piezo1‐linked impairment of insulin secretion (Homo‐Delarche et al. 2006; Nagy et al. 2018; Johansen et al. 2024). Islet inflammation in T2D is largely innate‐immunity driven, with increased intra‐islet macrophages that shift toward a proinflammatory M1‐like phenotype under metabolic stress (Ehses et al. 2007; Richardson et al. 2009), rather than adaptive, antigen‐specific insulitis characteristic of T1D. While hyaluronan accumulation in early T1D may transiently elevate local stiffness in early disease, the defining structural event in T1D is peri‐islet basement membrane degradation, which enables immune access and contributes to β‐cell demise.

Although NOD mice have been essential for defining early ECM remodeling in autoimmune diabetes, several well‐documented interspecies differences limit direct comparison with human T1D. Female NOD mice develop diabetes with near‐universal penetrance compared to male mice under standard specific‐pathogen‐free housing conditions (MacLaren et al. 1989), however, incidence varies substantially across facilities due to differences in colony microbiota composition (Fernandez Trigo et al. 2024). Further, mice display insulitis patterns that differ substantially from the sparse, heterogenous immune infiltration observed in autoantibody‐positive and recent‐onset human donors (Foulis et al. 1986; In't Veld et al. 2007; Willcox et al. 2009; Wiberg et al. 2015; Higuchi et al. 1992; Picarella et al. 1993). Peri‐islet structure and endocrine architecture differ markedly between humans and mice (Irving‐Rodgers et al. 2008; Virtanen et al. 2008), influencing both endocrine cell interactions with the ECM inside the islet and immune cell interactions with the ECM barrier from the outside. These distinctions influence the timing and severity of ECM disintegration across species. While NOD mice exhibit extensive and synchronous patterns of early ECM loss, pre‐onset human T1D shows selective remodeling, most clearly collagen IV loss, with broad basement‐membrane disintegration becoming more evident in Stage 3 as presented earlier (Johansen et al. 2025; Irving‐Rodgers et al. 2008; Ziolkowski et al. 2012; Korpos et al. 2013). Whether additional components (e.g., laminin, nidogen, and perlecan) undergo similar early changes in humans remains unresolved as current human pre‐onset datasets are more limited than extensively characterized Stage 3 T1D samples; live tissue slices may enable real‐time understanding of ECM changes in distinct T1D stages, a technique recently employed to study β‐cell behavior under T1D‐milleau in human samples (Huber et al. 2025).

While these data support the long‐held concept that β‐cells are “victims” of the breakdown of ECM‐mediated protection, new evidence suggests that β‐cells participate in the disintegration of the ECM in T1D pathogenesis as well, as discussed in the next section.

### 2.3 Cellular Contributors of ECM Disintegration in T1D

**FIGURE 1:**
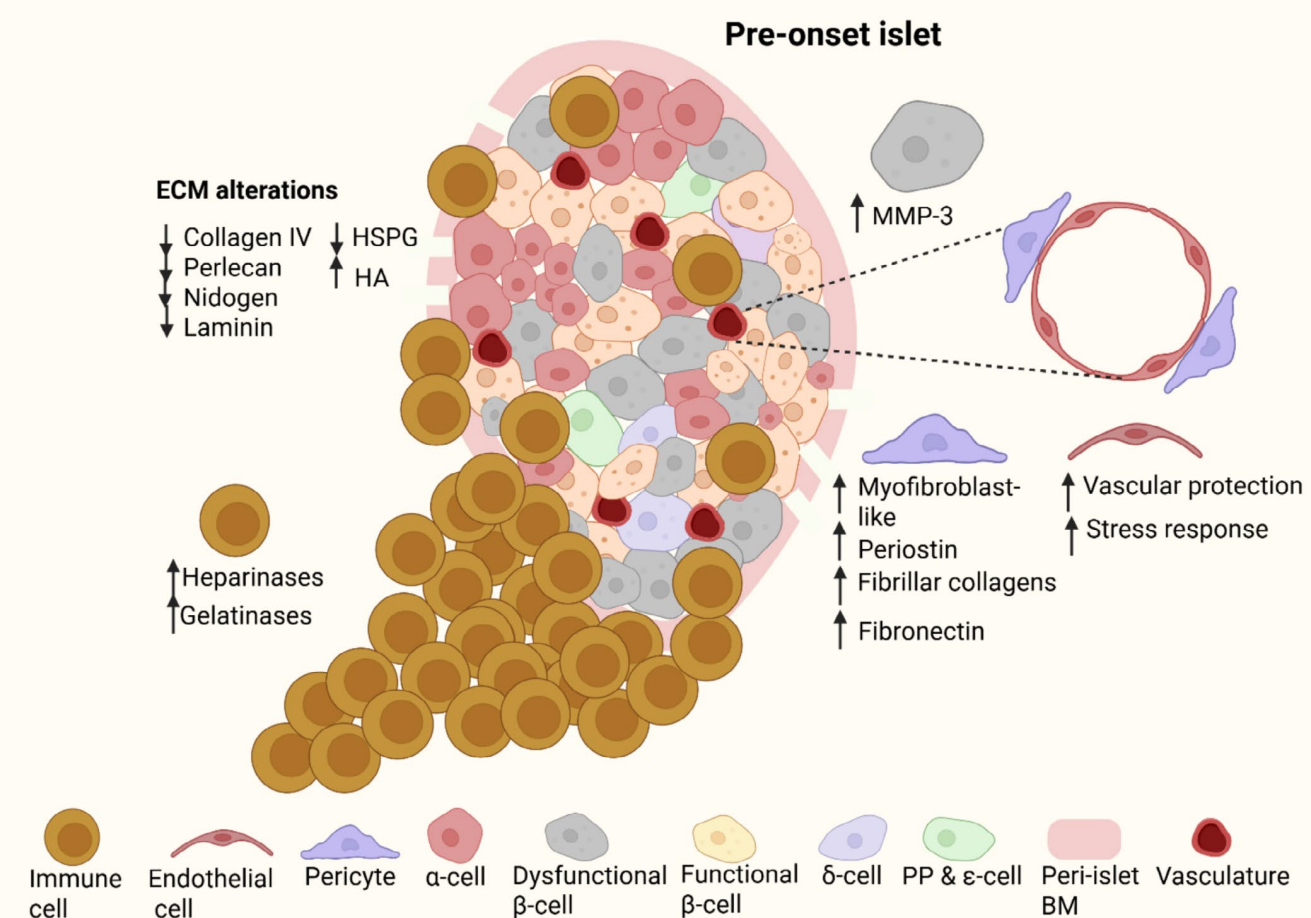
Cellular participants in pancreatic islet extracellular matrix collapse in pre‐onset T1D. Findings from both pre‐onset T1D human and NOD mice specimens were collated to summarize cellular contributors to peri‐islet ECM collapse. In pre‐onset islets, cells within the islet vicinity can release factors involved in ECM remodeling. Immune cells release increased levels of heparinases and gelatinases, while dysfunctional β‐cells can release matrix metalloproteinase 3 (MMP‐3). Pericytes lining the vasculature adopt a myofibroblast‐like status and produce high levels of periostin, fibrillar collagens, and fibronectin; findings linked to individuals with GADA‐positivity only. Vascular endothelial cells display an upregulation of vascular protection and stress response mechanisms. Pancreatic islet alterations in pre‐onset (Stages 1–2) T1D include alterations of extracellular matrix (ECM) components; loss of collagen IV, perlecan, nidogen, laminin, heparin sulfate proteoglycans (HSPG), and increased hyaluronan (HA). Degradation of the basement membrane (BM), as shown by dashed pink border and interstitial matrix (underneath cells), are also evident due to ongoing immune pressures. This decreases the number of functional β‐cells and promotes presence of β‐cells harboring dysfunctions. Adapted from (Atkinson and Mirmira 2023). Created in BioRender. Esparza, D. (2026) https://BioRender.com/vzf3m9u.

Matrix metalloproteinase‐3 (MMP‐3) degrades collagen IV and activates other MMPs, amplifying ECM remodeling during inflammation. Johansen et al. first reported increased MMP‐3 transcript and protein in mouse and cadaveric human islets exposed to a proinflammatory cytokine mix or chronic high glucose, mimicking T1D stress (Johansen et al. 2025). MMP‐3 was upregulated in insulin‐positive islet cells of NOD mice and in human pancreatic tissue from autoantibody‐positive, pre‐onset, and Stage 3 T1D individuals compared to their respective non‐diabetic controls. Consistent with the collagen degrading ability of MMP‐3, a loss of collagen IV was also evidenced in samples from pre‐onset and Stage 3 T1D individuals compared to nondiabetic controls (Johansen et al. 2025). In this same study, bulk RNA‐sequencing of cadaveric human islets exposed to pro‐inflammatory cytokine mix revealed upregulation of collagen‐catabolic genes (MMP‐1, MMP‐3, MMP‐10, MMP‐25, ADAMTS1, ADAMST4, and ADAMST9) and downregulation of collagens (COL4A5, COL26A1, and COL14A1) compared to untreated samples. Analogous to cancer cells that “prepare the soil” for metastasis, β‐cells actively condition the islet microenvironment, dismantling structural barriers and, in concert with stromal and vascular cells, signaling immune cells to infiltrate. These mechanisms are summarized in Figure 1 and further elucidated below.

Islet ECM components are secreted by both endothelial cells and pericytes, with their turnover regulated by intrinsic stromal cells, such as quiescent pancreatic stellate cells (PSCs) under physiological conditions. Recent single nucleus multiomic and spatial transcriptomic analyses revealed activated PSCs in pancreatic tissue from Stage 3 human T1D progressors (< 1 year disease duration), with these changes less pronounced in autoantibody‐positive, pre‐onset, individuals and absent in nondiabetic controls (Melton et al. 2025). Activated PSCs exhibited strong mRNA expression of collagens (COL1A1, COL1A2, COL3A1, and COL6A3), matrix proteolytic enzymes (MMP‐2 and LOX), as well as profibrotic growth factors and mediators (PDGFRA, FGF7, and SERPINE) (Melton et al. 2025). These signaling programs resemble fibrotic and ECM deposition processes reported in pancreatic ductal carcinoma (Kuninty et al. 2019; Biffi and Tuveson 2020; Shi et al. 2022). In contrast, islet endothelial cells primarily displayed an increased mRNA expression of genes associated with vascular protection and stress response (CLEC1A and PECAM1), and the hyaluronan degrading enzyme (HYAL2), with only modest mRNA expression of ECM‐related genes, such as COL81A and the proteoglycan modifier SULF2 in these same samples (Melton et al. 2025). These expression patterns align with endothelial cells under inflammatory or stress conditions associated with Stage 3 T1D reported in (Korpos et al. 2013; Richardson et al. 2023; Marei et al. 2025), where ECM components are still synthesized but matrix degradation and vascular remodeling to facilitate immune infiltration and angiogenesis are prioritized.

In a separate study, multimodal imaging revealed pericyte‐to‐endothelial cell density around islet capillaries was significantly reduced in the pancreata of individuals with Stage 3 T1D, but not in tissue from pre‐onset, GADA autoantibody‐positive only individuals, or in nondiabetic individuals (Mateus Gonçalves et al. 2023). Furthermore, RNA‐seq analysis from the Human Pancreas Analysis Program revealed pericytes/stellate cells within islets from individuals displaying only GADA autoantibody‐positivity switch toward a pro‐fibrotic, myofibroblast‐like state. This shift is marked by a significant upregulation of periostin (POSTN), fibrillar collagens (COL1A1, COL4A1, and COL4A2), and fibronectin (FN1) compared to levels observed in samples from Stage 3 T1D and nondiabetic individuals. Thus, pre‐onset human T1D may not be characterized by pericyte loss, but by a transcriptional shift toward a profibrotic, myofibroblast‐like state. Collectively, these changes underlie mechanisms that may contribute to vasomotor dysfunction and fibrotic remodeling during the early stages of islet autoimmunity.

Given the evidence above, stromal and vascular cells under a T1D‐like milieu during pre‐onset stages contribute significantly to ECM remodeling observed in Stage 3 T1D through coordinated processes of matrix deposition and degradation. In addition, studies in NOD mice and human pancreatic tissue sections indicate that activated immune cells are implicated in ECM degradation during T1D Stages 1–3, primarily through secretion of heparinases and gelatinases, which target fibrillar collagens I and III, elastin and fibronectin (Irving‐Rodgers et al. 2008; Ziolkowski et al. 2012; Korpos et al. 2013; Montgomery et al. 1993; Owen and Campbell 1999; Vaday and Lider 2000; Parish et al. 2013; Saunders et al. 2021). It is this iterative disintegration of the ECM by β‐cells and by immune cells which yields the description of the process as “chicken‐or‐egg”‐driven.

## 3 β‐Cell Dysfunction: Endoplasmic Reticulum and Golgi Apparatus Stress as Contributors

In the β‐cell, the ER and Golgi apparatus form the backbone of insulin biosynthesis and insulin granule exocytosis mechanisms. Although the precise initiating trigger of T1D remains unclear, stress responses during the pre‐onset period of T1D activate maladaptive pathways within the ER and Golgi, impairing (1) the insulin secretory pathway, and (2) increasing immune visibility (Aslamy, Oh, Ahn, et al. 2018; Aslamy, Oh, Olson, et al. 2018; Iida et al. 2023; Maestas et al. 2024; Mannering et al. 2005; Jin et al. 2011; McGinty et al. 2014; van Lummel et al. 2014; Xiang et al. 2015; Phelps et al. 2016; Bone et al. 2020; Isaacs et al. 2021). These mechanisms are described below.

### 3.1 β‐Cell Apoptosis and Identity Loss Are Linked to ER and Golgi Stress

Under conditions of heightened insulin demand, the unfolded protein response (UPR) pathway helps maintain ER homeostasis by slowing protein synthesis, increasing chaperone production, and promoting the degradation of misfolded proteins in β‐cells (Nakato et al. 2015; Pandey et al. 2019). However, chronic ER stress causes a reduced expression and activity of sarco/endoplasmic reticulum calcium ATPase (SERCA) in β‐cells, leading to an imbalance in Ca^2+^, impairing proinsulin processing and insulin secretion. This disruption promotes β‐cell dysfunction and eventual death through the activation of apoptotic pathways (Iida et al. 2023).

**FIGURE 2:**
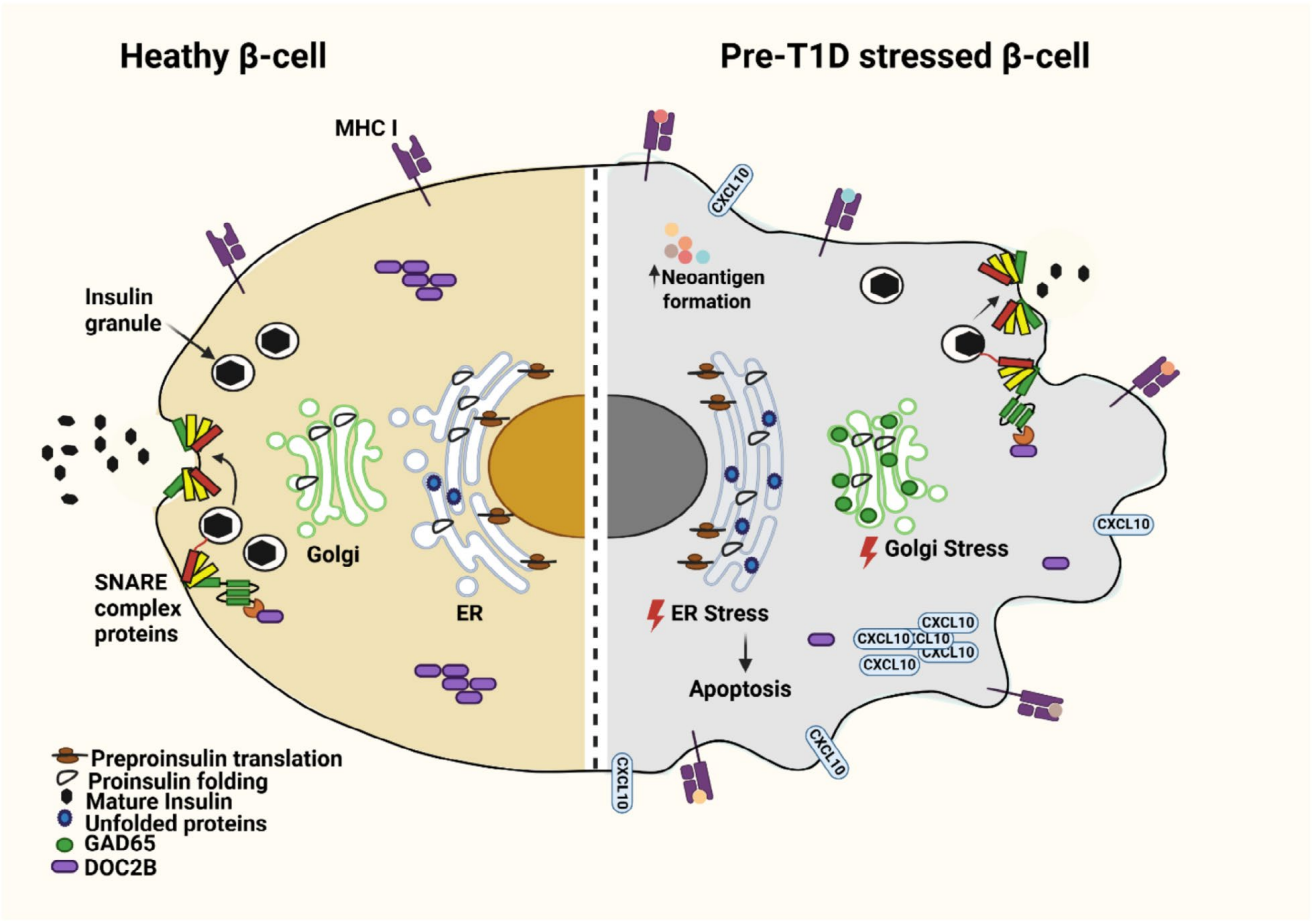
Secretory pathway alterations contribute to β‐cell vulnerabilities in pre‐onset T1D. In healthy islets, β‐cells maintain insulin secretion through a properly functioning secretory pathway, including efficient insulin exocytosis mediated via SNARE proteins and the SNARE regulatory protein, DOC2B. During pre‐onset T1D stages (T1D Stages 1–2), pro‐inflammatory cytokines trigger endoplasmic reticulum (ER) and Golgi apparatus stress, and vesicle trafficking errors in β‐cells. These disruptions lead to increased chemokine ligand 10 (CXCL10) generation, intracellular neoepitope formation for presentation in major histocompatibility complex (MHC) I molecules, GAD65 accumulation in the Golgi, enhanced MHC I expression on the surface of cells, and reduced DOC2B levels. In the nucleus of pre‐T1D β‐cells, increased expression of Golgi stress (COPZ2, KDELR1, CREB3, ARF4, ATF3, COG6, and GOSR2), as well as closed conformation of the insulin gene and open conformation of RABEPK (not shown in figure) have been noted. Created in BioRender. Esparza, D. (2026) https://BioRender.com/ltfalhc.

Recent studies on Golgi stress suggest it may be implicated in the loss of β‐cell identity and impaired function in T1D. Single‐cell RNA sequencing by Maestas et al. revealed that brefeldin A (BFA) exposure, an inhibitor of ER‐to‐Golgi trafficking, led to the loss of genes associated with regulation of insulin secretion and β‐cell development (Maestas et al. 2024). Chromatin remodeling events in response to BFA included a closed conformation of the *INS1* gene, which could hinder insulin production, and an open conformation of the *RABEPK*, a gene involved in endosomal–TGN transport. Whether Golgi stress is a consequence of ER stress or an independent malfunction in pre‐diseased β‐cells remains unresolved. These mechanisms are summarized in Figure 2 and further discussed below.

### 3.2 ER Stress, Loss of DOC2B, and Impaired Insulin Secretion: Interdependencies?

In islet β‐cells, DOC2B is an essential regulator of both phases of insulin exocytosis (Ramalingam et al. 2012). At present, there are no β‐cell specific DOC2B knockout models, but DOC2B haploinsufficiency in mice increases β‐cell vulnerability to diabetogenic injury in the multiple‐low‐dose streptozotocin (STZ) model (Aslamy, Oh, Olson, et al. 2018), which engages in inflammatory pathways to simulate those activated in human T1D. This phenotype is consistent with the concept of reduced DOC2B compromising β‐cell resilience even though it does not independently trigger autoimmune disease.

DOC2B protein deficiency is a feature of Stage 3 T1D human islet β‐cells, and cadaveric human islets and rodent β‐cell lines exposed to pro‐inflammatory cytokines (Aslamy, Oh, Ahn, et al. 2018; Aslamy, Oh, Olson, et al. 2018). Cytokine exposure induces oxidative and ER stress marker expression [iNOS, CHOP, phosphorylated (p)Eif2a] and apoptosis markers [PARP and cleaved caspase 3 (CC3)] (Aslamy, Oh, Ahn, et al. 2018; Aslamy, Oh, Olson, et al. 2018), suggesting that inflammation‐driven stress contributes to DOC2B downregulation. CXCL10, a chemoattractant for auto‐aggressive lymphocytes, is expressed by stressed β‐cells before detectable insulitis and is elevated in the serum of pre‐onset individuals (Nicoletti et al. 2002; Schulthess et al. 2009; Sarkar et al. 2012; Bender et al. 2016). Partial loss of DOC2B increases β‐cell susceptibility to stress‐induced destruction in multiple low‐dose streptozotocin‐treated mice and is associated with enhanced CXCL10 expression (Bhowmick et al. 2025). Meanwhile, β‐cell specific DOC2B overexpression protects mice from STZ‐induced glucose intolerance and β‐cell apoptosis, indicating enhanced resilience under diabetogenic stress (Aslamy, Oh, Olson, et al. 2018).

Furthermore, earlier studies suggested DOC2B enrichment protects β‐cells from thapsigargin‐induced ER stress, where its tandem C2 domain and its Ca^2+^ binding capacity were sufficient to confer protection alike that of the native/wild‐type form of DOC2B (Aslamy, Oh, Olson, et al. 2018). In cytokine‐stressed human islets and INS‐1832/13 β‐cells, DOC2B enrichment attenuates cytokine‐induced CXCL10 expression and protein levels of IKKβ and STAT1, as well as the phosphorylated form of STAT1, suggesting that DOC2B could constrain these inflammatory pathways (Bhowmick et al. 2025). Given that DOC2B enables STX4 activation in β‐cells (Aslamy, Oh, Olson, et al. 2018), where STX4 modulates IKKβ‐IκBβ‐NFκB (Oh et al. 2018; Veluthakal et al. 2021), and that DOC2B associates with IKKβ and STAT‐1 in cell‐free systems (Bhowmick et al. 2025), DOC2B may regulate these pathways through either STX4‐dependent or ‐independent interactions. Pre‐onset young NOD mice (7‐week‐old, female) show a 90% reduction in DOC2B protein, indicating that DOC2B loss in islets is an early event in NOD disease progression (Aslamy, Oh, Ahn, et al. 2018). These findings suggest that DOC2B supports β‐cell resilience, and its deficiency may contribute to stress‐mediated β‐cell dysfunction and immune influx in T1D by promoting ER stress and UPR response, as well as CXCL10 expression. However, the rapid and synchronous disease course in female NOD mice indicates that the timing of DOC2B downregulation may not fully reflect the more heterogeneous progression seen in early human T1D.

### 3.3 ER and Golgi Stress Induce Antigen Presentation in β‐Cells

Chronic or maladaptive ER stress contributes to T1D onset and progression through antigen presentation and immune cell activation (Mannering et al. 2005; Jin et al. 2011; McGinty et al. 2014; van Lummel et al. 2014; Xiang et al. 2015; Phelps et al. 2016; Rondas et al. 2015). Prolonged ER stress promotes the release of post‐translationally modified (PTM) proteins, which enables processing and presentation on MHC I molecules as neoantigens, activating CD4‐positive T cells (Mannering et al. 2005; McGinty et al. 2014; van Lummel et al. 2014; Rondas et al. 2015). ER stress also disrupts the proper palmitoylation of GAD65, a modification required for its translocation from the TGN to peripheral vesicles (Phelps et al. 2016). The abnormal accumulation of GAD65 at the Golgi membrane increases its presentation, leading to the development of autoreactive CD8‐positive T cells against GAD65 (Jin et al. 2011; Phelps et al. 2016).

In addition to chronic ER stress, Golgi dysfunction is emerging as a key amplifier of β‐cell immunogenicity in T1D. Using an integrative in silico approach that combined publicly available RNA‐seq and Microarray data with experimentally generated datasets, Bone et al. (2020) identified differential expression of Golgi‐associated genes in islets from T1D donors and in cadaveric human islets exposed to proinflammatory cytokines (IL‐1β and IFN‐γ) or BFA. Notably, genes related to MHC I antigen presentation and ER‐to‐Golgi transport, such as COPZ2 and KDELR1, exhibited differential expression patterns in T1D islets. Meanwhile, BFA‐treated human islets exhibited increased expression of canonical Golgi stress markers (CREB3, ARF4, and ATF3) along with components critical for Golgi integrity and vesicular trafficking (COG6 and GOSR2). These findings suggest that Golgi stress may be a contributing factor to β‐cell dysfunction in T1D via increased antigen presentation. Beyond impairing intracellular processes, ER and Golgi stress also reprogram the β‐cell secretome. This topic is discussed next.

## 4 Stress‐Induced Remodeling of the β‐Cell Secretome: Signals Preceding Disease Onset

### 4.1 The β‐Cell Secretome Under Normophysiological Conditions

Direct assessment of the β‐cell secretome within intact pancreatic tissue provides the most physiologically relevant context for understanding β‐cell function and its interaction with the microenvironment (Panzer et al. 2020; Panzer and Caicedo 2023). Due to the scarcity of pancreatic tissue from both nondiabetic and T1D individuals, the field has relied on isolated islets from cadaveric donors and β‐cell lines as proxies for assessing β‐cell secretory activity. These models have been pivotal in revealing how β‐cells support the islet microenvironment under normophysiological conditions through secretion of soluble molecules that help maintain the vasculature, innervation, β‐cell mass, and insulin secretion (Brissova et al. 2006; Inoue et al. 2002; Christofori et al. 1995; Moin et al. 2012; Ryaboshapkina et al. 2022; Petrocchi‐Passeri et al. 2015; Hakonen et al. 2018). For example, insulinotropic peptides derived from neuroendocrine protein VGF, such as TLQP‐62 and neuroendocrine regulatory peptide (NERP)‐2, have been detected in conditioned media (CM) from rodent islets cultured ex vivo or β‐cell lines (Moin et al. 2012; Petrocchi‐Passeri et al. 2015).

Recent proteomic profiling has enabled unbiased characterization of additional classes of molecules in CM derived from non‐diabetic human islets cultured ex vivo *and* from human EndoC‐βH1 cells under normophysiological conditions (Pinheiro‐Machado et al. 2025; Ryaboshapkina et al. 2022). For instance, human islet CM contains proteins involved in glucose metabolism, PI3K/AKT and MAPK signaling, ECM organization and collagen assembly, vascular processes, and actin cytoskeleton organization (Pinheiro‐Machado et al. 2025). Proteomics analysis of CM from EndoC‐βH1 cells further identified signal peptide‐containing factors (INS, IAPP, CHGA, CPE, and PCSK9) as well as precursors of bioactive peptides implicated in insulin secretion and β‐cell health (Ryaboshapkina et al. 2022).

Beyond soluble factors, the cellular secretome also includes membrane‐encapsulated nanoscale structures that contain a diverse array of molecules, known as EVs (Hendrix et al. 2023). EVs are released by almost all cell types, including β‐cells, and serve as key mediators of intercellular crosstalk, influencing local and systemic processes, such as glucose metabolism [reviewed in (Chidester et al. 2020; Veluthakal et al. 2024)]. They range in size from approximately 30 to 10,000 nm and can be broadly classified by biogenesis (e.g., exosomes, microvesicles, and apoptotic bodies) or functional context (e.g., oncosome and migrasome) (Hendrix et al. 2023; Eguchi et al. 2020; Huang et al. 2019). Their cargo includes membrane proteins (e.g., tetraspanins CD9, CD63, and CD81), luminal proteins (e.g., TSG101, alix, and syntenin), and various nucleic acids, lipids, and metabolites (Teng and Fussenegger 2021).

In our studies, we applied a single EV analysis approach, Single Extracellular VEsicle Nanoscopy, to profile EVs released by human islets and β‐cell lines (MIN6, INS‐1832/13, EndoC‐βH1) under basal conditions (Esparza et al. 2024). We identified distinct EV properties; largely circular EVs had average sizes 78–92 nm, and on average 8–14 tetraspanin CD81, CD63, and CD9 molecules per vesicle. These findings underscore that even at basal states, EV composition and morphology vary across β‐cell models. Observed differences in EV properties could reflect cells of origin, including distinct species of origin, differentiation state, and culture conditions.

EVs derived from islets or β‐cells under basal conditions can transfer functional components to recipient β‐cells and other islet resident cells, such as phagocytes and endothelial cells, supporting processes such as insulin secretion, β‐cell survival, and vascular integrity under normophysiological (basal) and metabolic stress conditions (Figliolini et al. 2014; Vomund et al. 2015; Mandal et al. 2020). To provide context, under basal conditions, autoantigens (tetraspanin 7, GAD65, IA‐2, proinsulin/insulin, ZnT8), surface proteins (GLUT2, integrins, TNFR1/3, Syntaxin 1A), programmed death ligand 1 (PD‐L1), protein disulfide isomerase (PDI), among other proteins, as well as RNA species (miR‐375, miR‐483, miR‐21, miR‐126, miR‐423, and miR155) have been detected in β‐cell EVs (Sheng et al. 2011; Palmisano et al. 2012; Guay et al. 2015; Cianciaruso et al. 2017; Lakhter et al. 2018; Javeed et al. 2021; Dekkers, Lambooij, et al. 2024; Rao et al. 2025; Syed et al. 2026; Mandal et al. 2020; McLaughlin et al. 2016; Hasilo et al. 2017; Krishnan et al. 2019; Dickerson et al. 2020; Tesovnik et al. 2020), with largely overlapping detection in islet EVs. In our work, we identified DOC2B as an additional cargo under basal conditions (Esparza et al. 2024). Given the homeostatic roles of both soluble factors and EVs, the next section examines how T1D‐like conditions alter the β‐cell secretome, including EV cargo, and how these changes may influence cell‐to‐cell interactions and disease progression.

### 4.2 The β‐Cell Secretome Under T1D‐Like Conditions

Under chronic T1D‐like stress, the human islet secretome is modified. For example, pro‐inflammatory cytokine‐treated human islets have been shown to release robust levels of soluble ER chaperones, ECM organization remodeling factors, complement activation, and immune signaling molecules (Pinheiro‐Machado et al. 2025), which may set the stage for subsequent immune infiltration. In the context of β‐cell EV signature changes, many features detected under basal conditions persist during T1D‐like stress, and some appear enriched in the latter. However, bulk EV analysis (e.g., immunoblot, ELISA, proteomics, and transcriptomics) aggregates signals across diverse vesicles and cannot fully resolve whether enrichment reflects increased molecule loading per EV or shifts in EV subtype composition. For example, CXCL10, gp96, and calreticulin are reported enriched in β‐cell or islet‐derived EVs upon proinflammatory cytokine exposure, but these changes also coincided with increased total EV numbers (Cianciaruso et al. 2017; Javeed et al. 2021), highlighting the interpretive ambiguity inherent to bulk EV measurements.

As shown in Table 1, findings on EVs released under cytokine exposure vary widely across studies due to differences in β‐cell models (MIN6, NIT‐1, EndoC‐βH1, rodent islets, and human islets); cytokine combinations and their concentrations/treatment time; EV isolation and quantification methods; and the activation state and identity of recipient immune cells (Cianciaruso et al. 2017; Lakhter et al. 2018; Javeed et al. 2021; Dekkers, Lambooij, et al. 2024; Rao et al. 2025). These variables influence both EV release and EV cargo: shorter exposures (~24 h) tend to have little effect, whereas prolonged treatments (48 h or multi‐day media changes) tend to result in increased EV release (Cianciaruso et al. 2017; Lakhter et al. 2018; Javeed et al. 2021; Rao et al. 2025). These observed differences could be in part due to decreased cell viability with longer cytokine exposure. Importantly, nanoparticle tracking analysis (NTA), often used to evaluate EV concentration, quantifies all nanoparticles within the sample and cannot distinguish EVs from other particles such as protein aggregates (Maas et al. 2015), which complicates interpretation of true changes in EV abundance. Meanwhile, ER stress induced by HSPA5 knockdown did not impact the number of released EVs (Dekkers, Lambooij, et al. 2024). On the recipient side, immune responses to β‐cell EVs also differ across contexts, with basal EVs capable of activating innate and adaptive immune cells and cytokine‐stressed EVs often amplifying these effects, although not uniformly across systems (Cianciaruso et al. 2017; Javeed et al. 2021; Rao et al. 2025; Vomund et al. 2015; Dekkers, Pu, et al. 2024). For example, compared to peripheral‐blood mononuclear cells (PBMCs) from non‐diabetic individuals, PBMCs from individuals with T1D show stronger activation in response to EVs from basal islets (Rutman et al. 2018). Together, these methodological and biological sources of heterogeneity limit cross‐study comparisons and caution against assuming consistent EV‐mediated immune activation under inflammatory conditions.

**TABLE 1:** EV‐mediated cargo transfer and its role in autoimmune responses in recipient cells relevant for T1D progression.

EV source (cell type)	Condition of EV donor cells	Recipient immune cells	Immune response and key findings	References
MIN6 (murine β‐cells)	Basal	In vitro cultured NOD mouse splenocytes and APCs	EVs upregulated MHC II and costimulatory molecules (CD80, CD86, ICAM‐1) and secretion of IL‐6 and TNF‐α from in APCs compared to EV‐free media	Sheng et al. (2011)
MIN6 (murine β‐cells)	Inflammatory	In vitro cultured BMDMs and CD8‐positive T cells (splenic) from C57BL/6	EVs from IL‐1β, TNF‐α and IFN‐γ increased TNF‐α and IL‐6 secretion from BMDM, MHC I/II presentation, and chemotaxis. EVs also induced CD8‐positive T cell activation and cytotoxic potential, as compared to EVs from untreated cells or media only (no EVs)	Javeed et al. (2021)
NIT‐1 (murine β‐cells)	PD‐L1 over‐expression	In vitro cultured NOD splenocytes	PD‐L1 EVs increased IFN‐γ and granzyme production of splenocytes; Inhibition of proliferation and activation of CD8‐positive T cells compared to EVs from vehicle treated cells	Rao et al. (2025)
EndoC‐βH1 (human β‐cells)	ER‐stress induced by HSPA5 knockdown	In vitro cultured ND human PBMC monocytes	shHSPA5 EVs significantly increased expression of integrin CD11b, HLA‐DR, CD40, and CD86 in monocytes and secretion of IL‐1β and IL‐6 compared shCTRL EVs	Dekkers, Lambooij, et al. (2024)
Murine islet β‐cells	Non‐inflammatory (NOD.*Rag1−/−*mice); high glucose (25 mM) and ER‐stress induced by Thapsigargin	APCs from NOD.*Rag1−/−*, NOD, C57BL/6 mice, and CD4‐positive T cell clones (11T‐3 and 8F10)	APCs presented peptides to 8F10 and 11T3 CD4‐positive cells leading to their activation; high glucose and Thapsigargin further increased vesicle transfer and T cell activation compared to 5 mM or vehicle conditions	Vomund et al. (2015)
Rat islets	Inflammatory	In vitro cultured HLA‐DR4^+/+^ transgenic mice‐derived BMDCs	EVs from IL‐1β and IFN‐γ‐treated rat islets increased secretion of TNF‐α and IL‐6 from BMDCs compared to EVs from untreated cells	Cianciaruso et al. (2017)
Human islets	Basal	In vitro cultured T1D human PBMC monocytes and B cells	EVs induced proliferation and activation of monocytes and B cells; B cells produced GAD65 autoantibody, with higher effects than non‐diabetic donor PBMCs	Rutman et al. (2018)

*Note:* Inflammatory stress indicates pre‐treatment of cells or islets with a combination of pro‐inflammatory cytokines (IL‐1β, TNF‐α, anf IFN‐γ). Sh = short hairpin RNA knockdown. CTRL = control.

Abbreviations: BMDC, bone marrow‐derived dendritic cells; BMDM, bone marrow‐derived macrophages; IFN‐γ, interferon γ; IL‐1β, interleukin 1β; IL‐2, interleukin‐2; IL‐6, interleukin 6; Immune cells APC, antigen presenting cells; MHC, major histocompatibility complex; NOD, nonobese diabetic mice; PD‐L1, programmed death‐ligand one; TNF‐α, tumor necrosis factor α.

These limitations further highlight the need for single EV analysis approaches to robustly characterize β‐cell EVs and uncover meaningful molecular signatures. Toward this, Rao et al. employed single‐particle interferometric reflectance imaging sensor (Exoview) to assess PD‐L1 levels on individual EVs from EndoC‐βH1 and human islets exposed to IFN‐α or IFN‐γ (Rao et al. 2025). They observed significant PD‐L1 enrichment in CD63‐, CD9‐, and CD81‐positive EV subpopulations without changes in total EV number, with the most pronounced increase in CD81‐positive EVs from EndoC‐βH1 under cytokine treatment, highlighting a unique EV subpopulation associated with β‐cell stress.

### 4.3 Accessing the Plasma Secretome as a Source of β‐Cell Biomarkers for Pre‐Onset T1D, and the Emergence of DOC2B

While autoantibody‐positivity remains the conventional approach for monitoring individuals at risk for T1D development, emerging evidence suggests that circulating factors in plasma may be value‐added for T1D risk stratification. Circulating factors have been linked to a β‐cell source under conditions of stress or apoptosis. Whether secreted as free molecules or packaged within EVs, these factors may capture intracellular β‐cell responses that might turn maladaptive (e.g., inefficient prohormone processing, issues with protein folding, transcriptional irregularities ongoing with ER stress or apoptosis) before overt dysfunction. For example, prediabetic NOD mice display increased serum or plasma proinsulin‐to‐C‐peptide (PI:C‐peptide) ratios (Tersey et al. 2012; Watkins et al. 2016), elevated PDIA1 (Syed et al. 2023), reduced levels of DOC2B protein (Aslamy, Oh, Ahn, et al. 2018), higher SASP (Thompson et al. 2019; Midha et al. 2021), and higher unmethylated *INS* DNA (Husseiny et al. 2014). These changes in serum and plasma are largely correlated with changes noted in islets from these same mice. Collectively these factors represent promising candidates for biomarker development as they capture early β‐cell stress and may serve as more accurate markers to predict disease risk as discussed below.

Indeed, patterns of factor release observed in prediabetic NOD mice are likewise evident in autoantibody‐positive individuals at risk for T1D development. At pre‐onset stages, the PI:C‐peptide ratio rises progressively, with greater changes in those with multiple autoantibodies or dysglycemia (Røder et al. 1994; Truyen et al. 2005; Sims et al. 2016). Higher ratios have been associated with rapid progression from Stage 2 to Stage 3 (Sims et al. 2023). Importantly, the PI:C‐peptide ratio represents a validated biomarker supported by replicated findings across multiple independent cohorts (Røder et al. 1994; Truyen et al. 2005; Sims et al. 2016, 2023).

Human pancreatic tissue from both autoantibody‐positive pre‐onset and onset T1D individuals shows abundant PDIA1 in insulin‐positive islet cells (Syed et al. 2023). In contrast, specimens from individuals with disease onset lacking insulin‐positive cells exhibit reduced PDIA1, similar to levels observed in non‐diabetic controls (Syed et al. 2023). Although PDIA1 has not been assessed in pre‐onset plasma, levels are elevated in T1D plasma compared to non‐diabetic controls (Syed et al. 2023), warranting further study.

Previous work indicates reduced DOC2B protein levels in circulating blood‐derived platelets from individuals with Stage 3 and Stage 4 T1D (Aslamy, Oh, Ahn, et al. 2018), a finding reproducible using plasma from two additional clinical trial cohorts (Esparza et al. 2025). Preliminary, non‐peer‐reviewed data from our group (Esparza et al. 2025) further suggest that lower plasma DOC2B levels in normoglycemic, pre‐onset (Stage 1–2), autoantibody‐positive individuals who later progressed to T1D may precede measurable changes in random C‐peptide and HbA1c, whereas non‐progressors maintained stable DOC2B levels. While tempting to speculate that this DOC2B loss could be reflective of stress occurring in β‐cells, in the absence of data from tissue‐specific knockout models, the β‐cell biomarker specificity remains incomplete. Toward this goal, DOC2B levels were evaluated in EVs from distinct cultured cell types known to express DOC2B; compared to EVs from cultured myotubes, EVs from cultured β‐cells had a higher DOC2B content (over whole cell lysates), while cultured neuronal‐like cells did not release appreciable DOC2B in the EVs (Esparza et al. 2024). The tandem C2 domain of DOC2B promotes packaging of DOC2B into EVs in β‐cells. These findings suggest that, in vitro, β‐cells (relative to other tested cell types) may preferentially package DOC2B into secreted EVs over soluble protein. We anticipate that emerging technologies will enable future in vivo quantification of β‐cell–derived DOC2B, in both soluble and EV forms, across healthy and pre‐onset T1D states.

**FIGURE 3:**
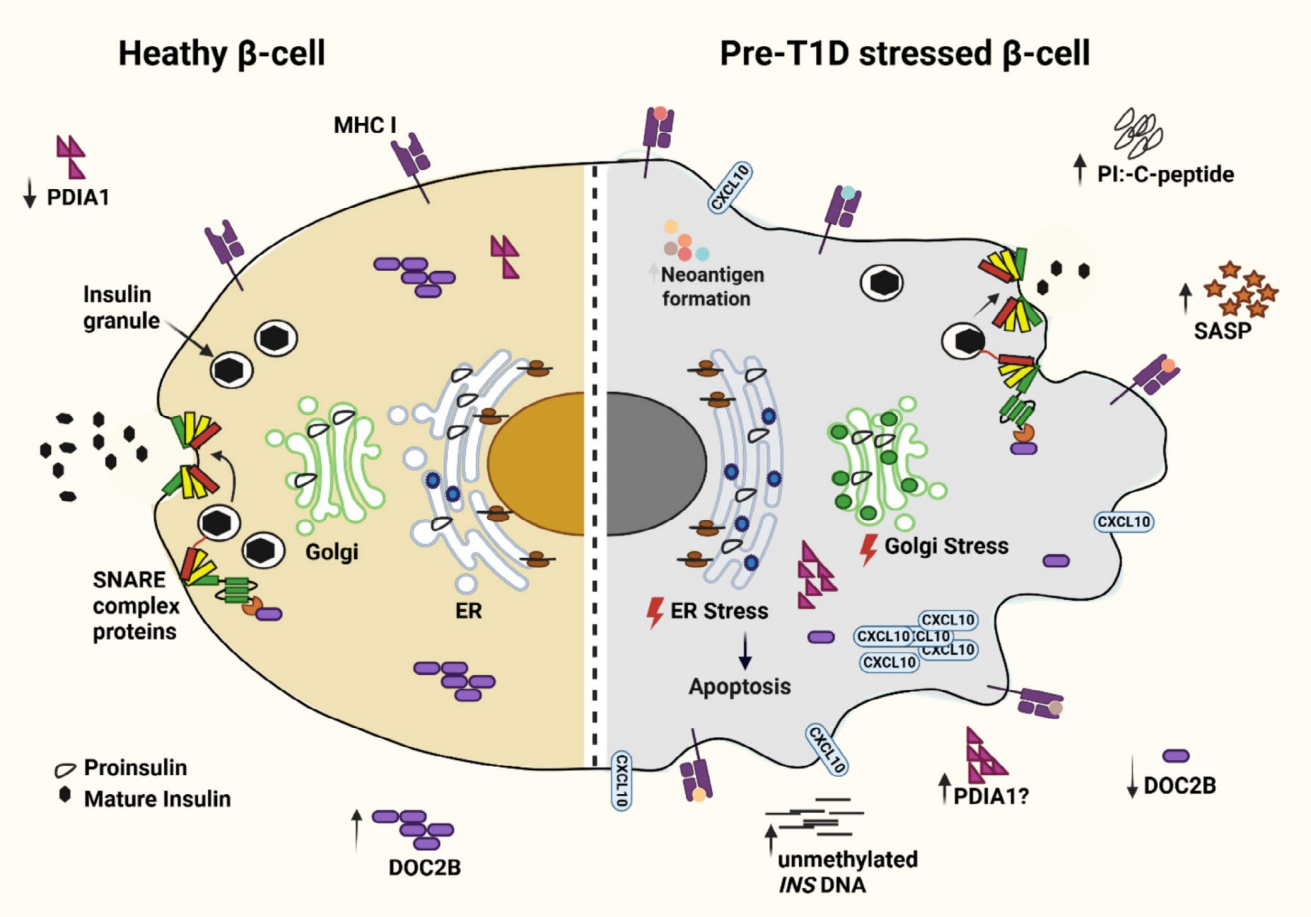
The secretome of stressed β‐cells in pre‐onset T1D as a source of biomarkers with clinical utility. In healthy islets, β‐cells maintain a secretome enriched in insulin and DOC2B, and low levels of protein disulfide isomerase A1 (PDIA1), reflecting normal ER and Golgi function. During pre‐onset T1D stages (T1D Stages 1–2), ER and Golgi stress becomes evident, characterized by elevated proinsulin (PI):C‐peptide ratio, increased cell‐free unmethylated insulin (*INS*) DNA, potentially higher levels of PDIA1, and secretion of senescence‐associated secretory phenotype (SASP) factors, alongside markedly reduced DOC2B in the secretome. Adapted from (Atkinson and Mirmira 2023). Created in BioRender. Esparza, D. (2026) https://BioRender.com/ss47twd.

Beyond these secreted factors, β‐cell apoptosis and senescence generate distinct biomolecules released into the extracellular space. β‐cell apoptosis significantly elevates circulating levels of unmethylated *INS* DNA, a molecular signature observed in pre‐onset autoantibody‐positive individuals who display glucose intolerance, indicating that β‐cell death can precede hyperglycemia (Herold et al. 2015). Similarly, β‐cell senescence emerges as a major driver of secretome remodeling in T1D (Thompson et al. 2019; Midha et al. 2021). In these β‐cell studies, senescence was defined by the emergence of a SASP‐like transcriptional and secretory program rather than by cell‐cycle arrest, which is not applicable to post‐mitotic endocrine cells. Senescent insulin‐positive cells accumulate in the pancreatic tissue of autoantibody‐positive individuals before onset, as marked by increased CDKN1A (p21) and SERPINE‐1 expression (Thompson et al. 2019). Importantly, senescent β‐cells release SASP factors, including chemokines (CXCL10, CXCL2, CXCL8, and CXCL1) and MMPs (MMP‐2, MMP‐3, and MMP‐12) prior to demise (Midha et al. 2021). Beyond their utility as biomarkers of β‐cell stress, it is notable that SASP factors have emerged as therapeutic targets, as senolytic elimination of senescent β‐cells in NOD mice prevents diabetes onset (Thompson et al. 2019), broadening avenues for future intervention. Overall, these factors with translational biomarker potential are depicted in Figure 3.

In addition, plasma EVs derived from individuals with T1D are attractive sources of biomarkers of underlying β‐cell dysfunction, as they carry diverse RNA species, including miR‐21‐5p associated with β‐cell dysfunction, and in some studies, immune modulators such as PD‐L1 (Lakhter et al. 2018; Rao et al. 2025; Garcia‐Contreras et al. 2017). Intriguingly, significantly higher levels of PD‐L1 have been reported in plasma EVs from pre‐onset autoantibody‐positive individuals (Rao et al. 2025), highlighting their potential for early disease risk assessment.

In summary, β‐cell derived factors, including soluble mediators and EV cargo, are promising biomarkers, but their clinical utility will depend on validation and understanding of regulated release. For some candidates, such as DOC2B, it will also be important to determine whether changes in circulating levels reflect β‐cell‐enriched release or a broader systemic stress response given the multi‐tissue expression and stress‐induced downregulation of this protein. This possibility is supported by the detection of preproinsulin‐containing EVs, likely sourcing from β‐cells, in human plasma following a glucose bolus, compared to fasting conditions (Ghosh et al. 2024), highlighting the potential to capture β‐cell signatures in plasma.

## 5 DOC2B: A Master‐Regulatory Target for T1D Intervention?

### 5.1 Multiple Positive Regulatory Actions Yield Whole‐Body Glucose Homeostasis

In tandem with modulation of the immune system, most T1D‐focused interventions seek to regenerate, repair, or replace β‐cells. There is great logic in this β‐cell focused approach for T1D. Thinking outside the box, however, DOC2B carries potential to protect and promote β‐cell function and viability, and via its broader positive roles in non‐β‐cells, beyond the islet. DOC2B is required for exocytosis in multiple cells, principally for pancreatic islet β‐cell insulin release, neuronal neurotransmitter release, and glucose transporter localization to the plasma membrane of fat and muscle cells to facilitate glucose uptake (Ramalingam et al. 2012; Verhage et al. 1997; Carvalho et al. 2005; Groffen et al. 2010; Pang Zhiping et al. 2011; Yao et al. 2011; Gaffaney et al. 2014; Bourgeois‐Jaarsma et al. 2019; Zhang et al. 2019; Chatterjee Bhowmick et al. 2022; Yu et al. 2013). In islet β‐cells, DOC2B promotes insulin exocytosis by acting as a scaffolding protein that supports SNARE complex assembly or via interactions with radixin (ERM) protein (Chatterjee Bhowmick et al. 2022; Ramalingam, Lu, et al. 2014; Ramalingam, Oh, and Thurmond 2014). These interactions enhance insulin granule trafficking, docking and fusion with the plasma membrane in response to glucose stimulation. Global DOC2B heterozygous or homozygous knockout mice exhibit glucose intolerance, loss of biphasic insulin secretion in islets, and impaired peripheral insulin sensitivity (Ramalingam et al. 2012; Aslamy, Oh, Olson, et al. 2018). These findings highlight the systemic need for DOC2B for whole‐body glucose homeostasis. Coordinately, evidence from a mouse model of global whole‐body DOC2B enrichment reveals benefits to whole‐body glucose homeostasis, with particular enhancements to islet β‐cell and skeletal muscle functionalities (Ramalingam, Oh, and Thurmond 2014). Tissue‐specific inducible enrichment of DOC2B has distinct effects depending on the target cell type: β‐cell‐specific DOC2B enrichment boosts islet β‐cell function (Aslamy, Oh, Olson, et al. 2018), whereas skeletal muscle‐specific DOC2B enrichment boosts skeletal muscle function and insulin sensitivity (Zhang et al. 2019). These findings highlight the systemic need for, and benefit from enrichment of, DOC2B for whole‐body glucose homeostasis.

### 5.2 DOC2B Mechanisms: Lessons From Metastatic Cancer Cells for β‐Cell Resilience

Cancer cell invasion requires dynamic actin cytoskeletal remodeling, including the formation of protrusions such as filopodia, lamellipodia, and invadopodia that support cell migration and ECM degradation (Lim et al. 2026). Filopodia can contribute to ECM engagement in part by spatially organizing protease activity, such as membrane‐anchored MMPs at the cell‐ECM interface (Pratiwi et al. 2024). Work in invasive cancer cells has shown that loss of intracellular DOC2B significantly increases the number and length of filopodia and promotes aberrant intracellular actin remodeling, whereas DOC2B's overexpression suppresses these features (Bhat et al. 2022). DOC2B also influences EV biology in these systems, where its overexpression increases vesicle release and enriches EVs in metabolites such as phosphatidylinositol 4,5‐bisohosphate (PIP2), L‐palmitoycarnitine, cholesterol ester, and active β‐catenin (Eswaran et al. 2025). EVs from DOC2B‐overexpressing cells can reduce filopodia in recipient cells in a calcium‐dependent manner, illustrating both intracellular and EV‐mediated routes by which DOC2B regulates actin‐associated protrusive behavior.

Unlike highly migratory cancer cells, healthy β‐cells are generally non‐motile and do not form classical filopodia. Instead, they rely on intracellular regulated actin remodeling to support glucose‐stimulated insulin secretion, a process requiring phosphorylated DOC2B and ERM protein activation (Chatterjee Bhowmick et al. 2022). Under pro‐inflammatory cytokine exposure, however, β‐cells undergo aberrant cytoskeletal actin remodeling (Groen et al. 2021), and DOC2B expression is markedly reduced (Aslamy, Oh, Ahn, et al. 2018). Cytokines have also been reported to induce MMP‐3 expression and reduce collagen IV in insulin‐positive islet cells (Johansen et al. 2025). While the subcellular localization of MMP‐3 in stressed β‐cells remains unknown, cancer studies suggest that dysregulated actin remodeling can influence how proteases intersect with the ECM.

Taken together, these observations support a conceptual sequence in which cytokine‐induced DOC2B loss may shift β‐cells from regulated actin remodeling toward a stressed state that alters both EV release and their cargo content and cell‐matrix interface. In this context, actin instability could facilitate the access or activity of proteases such as MMP‐3 at the peri‐islet ECM, contributing to localized collagen IV loss, while in vitro, cytokine‐exposed β‐cells are known to release EVs containing immune cell‐stimulating cargo (Table 1). ECM degradation could weaken the physical barrier that normally restricts immune cell access and increase the exposure or visibility of stressed β‐cell ligands presented via EVs, which could lead to immune recognition and engagement as summarized in Table 1. Although each component of this pathway is supported by experimental evidence, the integrated DOC2B‐EV‐ECM mechanism remains hypothetical and will require direct testing.

**FIGURE 4:**
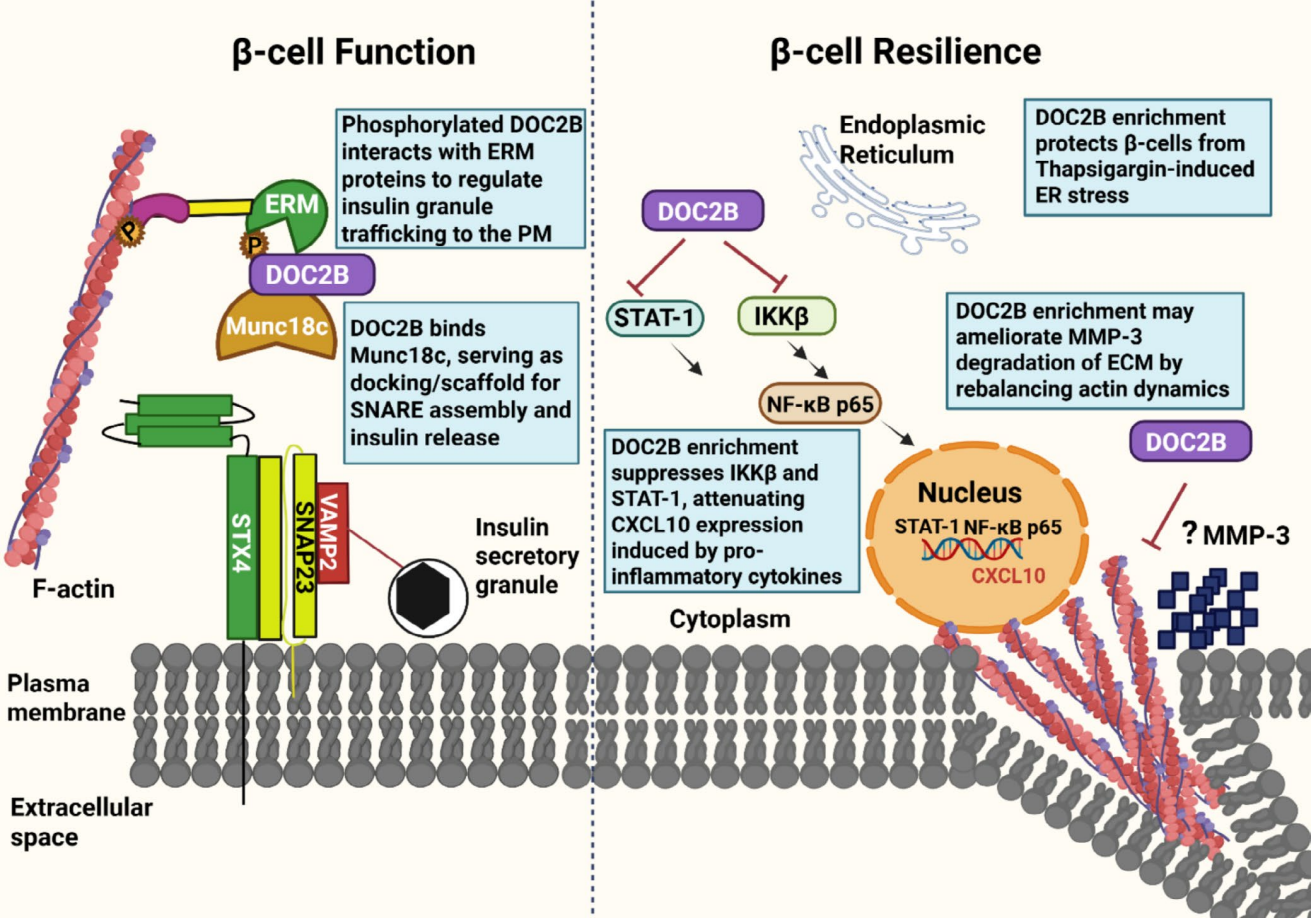
DOC2B as a potential avenue for T1D intervention. In pre‐onset T1D, DOC2B enrichment yields improved islet β‐cell function. This could be via mechanisms discerned in healthy β‐cells, such as (1) by acting as a scaffolding protein that interacts with Munc18c to facilitate SNARE complex assembly; and (2) through interactions with ERM protein Radixin (Chatterjee Bhowmick et al. 2022). In addition, under such conditions, it helps preserve β‐cell function by attenuating ER stress and cytokine‐induced expression of chemokine CXCL10 through negative regulation of transducer and activator of transcription one protein (STAT‐1) and IKKβ/nuclear factor kappa‐B (NF‐κB) p65. DOC2B enrichment may also aid in the mitigation of matrix metalloproteinase‐3 (MMP‐3) extracellular matrix degradation by regulation of maladaptive mechanisms governing hyperactive Actin polymerization. Created in BioRender. Esparza, D. (2026) https://BioRender.com/vx96lfw.

These parallels suggest that therapeutic strategies aimed at preserving or restoring DOC2B expression or stabilizing its actin‐regulatory balance could help maintain β‐cell integrity and prevent ECM degradation, thereby mitigating immune infiltration as shown in Figure 4.

## 6 Conclusions and Perspectives

While T1D ultimately leads to multi‐organ complications, the pathogenic signals arise when immune cells misinterpret islet β‐cell cues, partly due to MHC predispositions and environmental triggers. This positions pancreatic islets as the central hub for understanding molecular changes leading to disease progression. Recent advances in spatial transcriptomics, multi‐omics, and unbiased proteomics combined with access to samples from pre‐onset autoantibody‐positive individuals reveal that beyond immune misinterpretation, pancreatic β‐cells themselves may play a pivotal role in this process. Under chronic immune and metabolic pressures, their adaptive stress response becomes maladaptive, driving ECM degradation, aberrant antigen presentation, and altered secretory profiles. Therefore, identifying molecular regulators that govern this shift from adaptive to maladaptive responses is essential for understanding β‐cell dysfunction and designing targeted interventions.

Loss of DOC2B may represent one of several stress‐response vulnerabilities that exacerbate β‐cell dysfunction in T1D. DOC2B supports β‐cell functionality and resilience against pro‐inflammatory cytokines and ER stress, and its decline could further impair the ability of β‐cells to maintain secretory competence under autoimmune pressure. In cancer cells, DOC2B is suppressed to promote invasiveness, and its restoration reduces aberrant actin remodeling and limits the formation of actin‐rich protrusions, such as filopodia, which can spatially organize protease activity at the cell‐ECM interface. By analogy, reduced DOC2B in stressed β‐cells may negatively impact cytoskeletal organization and the local presentation or release of factors such as MMP‐3 that are associated with islet‐ECM interface; these specific mechanisms in human islets remain to be defined. In this sense, DOC2B is best viewed not as a singular factor that initiates defects but rather as a potential amplifier of β‐cell maladaptation during T1D progression.

While ER stress, distinct EV release, inflammation, and ECM remodeling occur in many diseases, their convergence in T1D creates a distinct microenvironment in which DOC2B loss may amplify these events. In this setting, reduced DOC2B may further modify the composition or release dynamics of β‐cell‐derived EVs. Because the proposed DOC2B‐EV‐ECM axis involves EVs interacting with or moving through the peri‐islet matrix, the physical properties of the ECM become directly relevant to how these particles can access immune cells. EV size can overlap with ECM nanopore size (Irvine et al. 2013), and steric hindrance can limit their passive movement, making ECM architecture an important determinant of EV accessibility. In the context of T1D, early β‐cell dysfunction coincides with an inflammatory milieu and basement membrane degradation. These conditions may allow EVs from stressed β‐cells to engage with antigen presenting cells, bone marrow‐derived dendritic cells, B cells, T cells, and splenocytes. Thus, the potential importance of a DOC2B‐EV‐ECM axis in the inflammatory, ECM‐permissive environment of T1D may be worthy of future investigation.

Harnessing these insights holds promise for the development of biomarkers and therapeutic strategies for T1D that may help mitigate β‐dysfunction and loss. Established plasma readouts, such as elevated PI:C‐peptide ratios, have been validated across multiple independent cohorts and consistently reflect β‐cell stress. Emerging candidates, including reduced DOC2B, may also report early β‐cell dysfunction before changes in random C‐peptide and HbA1c, but current evidence stems from preliminary, non‐peer‐reviewed work from our group and requires further validation.

Beyond its potential as a biomarker, DOC2B is emerging as a mechanistically informed target for preserving β‐cell function and potentially islet ECM integrity during T1D‐associated stress, both essential for GSIS. Correcting DOC2B levels may also benefit other organs that depend on its function, underscoring its systemic relevance. Notably, a mouse model of global DOC2B overexpression has not been reported to have any systemic adverse side effects. Indeed, these mice have enhanced glucose homeostasis (Ramalingam, Oh, and Thurmond 2014). Relatedly, DOC2B levels in platelets from islet cell transplant patients were substantially elevated within 30 days, correlating with their improved glycemia and no correlations with negative side effects (Aslamy, Oh, Ahn, et al. 2018), as well as in plasma from a larger cohort of islet cell transplant patients, in our recent non‐peer reviewed dataset (Esparza et al. 2025). Therefore, stabilizing or activating residual DOC2B protein may help maintain insulin granule fusion and β‐cell resilience under inflammatory stress. DOC2B mRNA levels are decreased in β‐cells under T1D‐related stress. If transcription is a limiting step, strategies that enhance endogenous DOC2B expression could therefore be beneficial. However, several challenges must be addressed for therapeutic translation. Although islets are highly vascularized, direct therapeutic delivery of nucleic acid or protein to β‐cells remains challenging (Melamed et al. 2023). Delivery strategies such as β‐cell‐tropic adeno‐associated virus (AAV) serotypes, lipid nanoparticles with islet‐targeting ligands, or local/intra‐pancreatic administration are active areas of development in the islet field (Melamed et al. 2023; Xiao et al. 2018). Whether islet targeting is required remains to be explored further, given that whole body enrichment of DOC2B in mice resulted in improved glucose homeostasis, linked to enhanced function in both islets and skeletal muscle (Francois et al. 2014).

In conclusion, linking β‐cell secretome readouts and communication cues to mechanistic pathways establishes a foundation for precision monitoring and actionable intervention, addressing a major gap in early detection and therapeutic targeting. Looking ahead, emerging platforms such as live pancreas tissue slide models will enable in situ investigations of molecular and secretome changes beyond insulin defects, derived from β‐cells, under T1D insults (Huber et al. 2025). This approach not only strengthens biomarker discovery but also supports FDA priorities for validating therapeutic targets in physiologically relevant contexts, paving the way for interventions, including candidates such as DOC2B, to preserve β‐cell integrity.

## Author Contributions

D.E., T.J.‐T., and D.C.T.: Conceptualization, writing – review and editing. D.E., T.J.‐T., D.C.T., and C.S.J.: Writing – original draft and editing. All authors have read and agreed to the published version of this manuscript.

## Funding

The author(s) declare financial support was received for research, authorship, and/or publication of this article. This work was supported by grants from the National Institutes of Health DK067912, DK112917, and DK102233 (DCT), fellowships from the Ford Foundation and National Institutes of Health DK102233‐05S1 (DE, DCT), and CUBRI fund (DCT, TJT), and Wanek Family Project Innovative Award (DCT, TJT).

## Conflicts of Interest

The authors declare no conflicts of interest.

## Data Availability

Data sharing not applicable to this article as no datasets were generated or analysed during the current study.
